# Childhood Depression: Relation to Adaptive, Clinical and Predictor Variables

**DOI:** 10.3389/fpsyg.2017.00821

**Published:** 2017-05-18

**Authors:** Maite Garaigordobil, Elena Bernarás, Joana Jaureguizar, Juan M. Machimbarrena

**Affiliations:** ^1^Department of Personality, Assessment and Psychological Treatments, Faculty of Psychology, University of the Basque CountrySan Sebastian, Spain; ^2^Department of Developmental and Educational Psychology, Faculty of Education, Philosophy and Anthropology, University of the Basque CountrySan Sebastián, Spain; ^3^Department of Developmental and Educational Psychology, University College of Teaching Training, University of the Basque CountryBilbao, Spain

**Keywords:** child depression, correlations, predictor variables, stress, resilience, self-concept, social skills, psychosomatic problems

## Abstract

The study had two goals: (1) to explore the relations between self-assessed childhood depression and other adaptive and clinical variables (2) to identify predictor variables of childhood depression. Participants were 420 students aged 7–10 years old (53.3% boys, 46.7% girls). Results revealed: (1) positive correlations between depression and clinical maladjustment, school maladjustment, emotional symptoms, internalizing and externalizing problems, problem behaviors, emotional reactivity, and childhood stress; and (2) negative correlations between depression and personal adaptation, global self-concept, social skills, and resilience (sense of competence and affiliation). Linear regression analysis including the global dimensions revealed 4 predictors of childhood depression that explained 50.6% of the variance: high clinical maladjustment, low global self-concept, high level of stress, and poor social skills. However, upon introducing the sub-dimensions, 9 predictor variables emerged that explained 56.4% of the variance: many internalizing problems, low family self-concept, high anxiety, low responsibility, low personal self-assessment, high social stress, few aggressive behaviors toward peers, many health/psychosomatic problems, and external locus of control. The discussion addresses the importance of implementing prevention programs for childhood depression at early ages.

## Introduction

Currently, depression is one of the mental illnesses that produces the greatest concern to health authorities. Its prevalence rate is increasing yearly, according to the World Health Organization (World Health Organization [WHO], 2016), affecting 350 million people worldwide. Depression has very negative consequences in all life areas (family, friends, work…) and it is an important public health problem, as well as leading to high health expenditure.

People who suffer depressive symptomatology in childhood and adolescence are more likely to suffer major depression or persistent depressive disorder (dysthymia) in adulthood. In addition, there is a possibility of suicide during major depression (American Psychiatric Association [APA], 2014). Therefore, it is necessary to continue deepening our study of the variables that can predict childhood depression. In this way, early preventive interventions could be implemented that would forestall further negative consequences.

Childhood depression requires special attention due to its influence on children’s comprehensive development and to its severe mid- and long-term consequences in adolescence and adulthood, for example, the risk of developing other mental pathologies. In fact, along with anxiety, depression is one of the most common mental health disorders in children and adolescents (World Health Organization [WHO], 2016).

Many studies alert about the high prevalence of depression at early ages. Studies carried out in schools with child population, using self-reports to appraise severe depression, indicated prevalence rates in Spain close to 4% at early ages (8–12 years) and somewhat higher rates (from 4.3 to 6.5%) in adolescence (for a review, see Jaureguizar et al., 2017). This prevalence is high and shows the need to identify variables that can predict depressive symptomatology at early ages to initiate actions to prevent these symptoms during childhood. Age is a variable that must be taken into account because the onset of major depressive disorders often occurs between 11 and 12 years of age, although the beginning of less severe depressive symptoms is observed mainly around 7–8 years (Del Barrio, 2000).

The quality of the interpersonal relations, anxiety, or self-esteem are some of the variables that appear in studies concerning childhood depressive symptomatology (Bernaras et al., 2013). Human beings need to belong to and interact with social groups because positive social relations are crucial for physical and psychological well-being. Specifically, children with peer relationship problems are more prone to suffer depressive symptoms (Kochenderfer and Ladd, 1996; Brendgen et al., 2002; Hames et al., 2013). Longitudinal studies confirm that difficulties in peer relationships predict childhood depression (Cole, 1991) and even depression in adolescence (Qualter et al., 2010; Katz et al., 2011). Katz et al. (2011) found that childhood loneliness predicted social impairment in adolescence and this, in turn, predicted depression in adulthood.

Anxiety is another variable closely related to depression. Lamers et al. (2011) observed that comorbidity of depressive and anxiety disorders was associated with a more traumatic childhood. In addition, in their study, they observed that, in 57% of the comorbid cases, anxiety preceded depression, and in 18%, depression preceded anxiety. By the other hand, Wu et al. (2016) found that, among other variables, self-esteem and depressive symptoms in childhood predicted high symptoms of social anxiety. In boys, anxiety itself is a good predictor whereas, in girls, the predictors of depression are anxiety, worry, and hypersensitivity (Kovacs and López-Durán, 2010).

Other studies focus on the relation between depressive symptomatology and self-esteem. It seems that children and adolescents who present depressive symptomatology have lower self-esteem (Orth et al., 2008; Bos et al., 2010). By the other hand, Geng-Feng et al. (2016) found that resilience (a positive trait that helps people face adversity and develop good personal adjustment) was negatively associated with depression and could be a positive trait to relieve the harmful effect of isolation.

Socialization contexts, especially, the school environment, deserve special attention due to the great relevance that school maladjustment acquires at this evolutionary stage and to its impact on personal adaptation/maladjustment. In line with this, recent studies have found connections between depression and low academic achievement (Jaureguizar et al., 2017).

Few studies have identified the predictive variables of child depression. Among them, we note the study of Wang et al. (2016), which confirmed problems of health and adaptation, interpersonal relations, and academic achievement as predictors of suffering depressive symptoms between ages 7 and 17. Reinfjell et al. (2016) observed that difficult infant temperament and parental depression predicted an increase in depressive symptoms, whereas social skills predicted their decrease. Lack of social support (Colman et al., 2014), childhood and adolescence behavior problems (Kosterman et al., 2010), adverse experiences in childhood (Poole et al., 2017), and low self-esteem (Babore et al., 2016) also predicted depression.

### Goals and Hypotheses

Taking into account previous studies, this research proposed two goals: (1) to study the relationships between self-assessed childhood depression and adaptive (social skills, self-concept-self-esteem, resilience, personal adaptation) and clinical variables (clinical disorder, school maladjustment, emotional symptoms, internalizing/externalizing problems, problem behaviors, childhood stress); and (2) to identify variables that predict childhood depression.

With these goals in mind, the study intends to verify whether children with many depressive symptoms, evaluated with a psychometric instrument, will also have: (1) high scores both in internalizing (withdrawal, somatization, anxiety) and externalizing problems (academic achievement), and school maladjustment (negative attitude to school); as well as (2) low self-concept-self-esteem, poor social skills (communication, cooperation, assertiveness…), and low resilience. In addition, this study attempts to confirm whether poor social skills, low self-esteem, and high level of anxiety predict childhood depression.

## Materials and Methods

### Participants

The sample was made up of 420 participants aged 7–10 years, 59.5% were between 7 and 8 years old (*n* = 250) and 40.5% were between 9 and 10 (*n* = 170), and there was a total of 224 boys (53.3%) and 196 girls (46.7%). The participants were selected from schools of the Basque Country (Spain), 53.6% from public schools (*n* = 225) and 46.4% from private/ concerted schools (*n* = 195). Public schools are state-funded and associated with middle and low socio-economic levels, whereas private schools are mainly funded by the parents’ economic contributions and are associated with high socio-economic levels. Participants studied third (*n* = 221, 52.6%) and fourth grade (*n* = 199, 47.4%) of Primary Education. Of the total sample, 81.9% (*n* = 344) had been born in the province of Gipuzkoa, 1.9% (*n* = 8) in other Spanish provinces, 5.2% (*n* = 22) were aliens and 11% (*n* = 46) did not answer that question. The sample was selected intentionally from the schools of Gipuzkoa, balancing public and private/concerted schools.

### Assessment Instruments

To measure the variables under study, we administered the Children’s Depression Scale (CDS-self-assessment) and another six assessment instruments with psychometric guarantees of reliability and validity (**Table 1**).

**Table 1:** Assessment instruments, assessed variables, tasks and psychometric data.

Instruments	Assessed variables	Task	Reliability
CDS-self-assessment, Child Depression Scale (Lang and Tisher, 1978; Seisdedos, 2003)	*Depression*	Self-report. 66 statements (e.g., “I often think that nobody cares about me”), rated on a 5-point scale according to the degree of agreement with the content of the sentence (1 = disagree strongly; 5 = strongly agree).	K-R 20 = 0.91. Study sample (α = 0.88)
BASC-S2. Behavior Assessment System for Children and Adolescents (Reynolds and Kamphaus, 1992; González et al., 2004)	Clinical scales Negative attitude to school Negative attitude to teachers Atypicality External locus of control Social stress Anxiety Depression Sense of incapacity Adaptive scales Interpersonal relations Relations with parents Self-esteem Self-confidence *Global Indices* *Clinical Maladjustment* *School Maladjustment* *Personal Adaptation* *Emotional Symptoms Index*	Self-report. 146 sentences of adaptive behaviors (e.g., “My parents are often proud of me”) and clinical symptoms (e.g., “I often have nightmares”). Respondents indicate the degree to which they can self-apply the content of the statement (thoughts, feelings, behaviors) on a True/False scale.	α = between 0.70 and 0.80 Study sample (α = 0.92)

SPECI. Screening for Children’s Emotional and Behavioral Problems (Teacher) (Garaigordobil and Maganto, 2012)	*Internalizing problems* Withdrawal Somatization Anxiety Infantile-dependence Thought problems Depression *Externalizing problems* Attention-hyperactivity Disruptive behavior Academic achievement Violent behavior *Emotional and behavior problems (EBP)*	Teacher’s assessment: evaluates 10 EBPs or categories through a series of illustrative examples of the problem (e.g., “Withdrawal: He/she is withdrawn and inhibited, prefers to be alone and seems isolated; not very active and reserved in his/her relation with others”). They indicate the frequency with which they observe these behaviors in each one of their students on a 3-point scale (0 = not at all, 1 = fairly, 2 = very much)	α = 0.82. Study sample (α = 0.89)

CAG. Cuestionario de Autoconcepto [Self-concept Questionnaire] (García, 2001)	*Global self-concept* Physical self-concept Social self-concept Intellectual self-concept Family self-concept Personal self-concept Sense of control	Self-report. 48 statements related to self-concept dimensions (e.g., “I like the way I am”). Respondents rate the degree to which they can self-apply the content of phrases on a 5-point scale (1 = Never, 2 = Not often, 3 = Don’t know, 4 = Often, 5 = Always).	α = 0.87. Study sample (α = 0.74)

SSIS. Social Skills Improvement System (Gresham and Elliott, 2008)	*Social skills* Communication Cooperation Assertiveness Responsibility Empathy Involvement/participation Self-control *Problem Behaviors* Externalizing Bullying Inattention-hyperactivity Internalizing	Self-report. 75 items (e.g., “I ask for information when I need it”). Should indicate degree of agreement with content through 4 response options: Not true, Not very true, Fairly true, Very true.	α = 0.80 to 0.90. Study sample: (α = 0.98 to 0.99)

RSCA. The Resiliency Scales for Children and Adolescents (Prince-Embury, 2008)	*Sense of competence* Optimism Self-efficacy Adaptability *Sense of affiliation* Self-Confidence Support Comfort Tolerance *Emotional reactivity* Sensitivity Recovery Alteration	Self-report. 64 statements (e.g., “I can adapt when they change plans”). Respondents indicate the frequency with which they have the thought, feeling or behavior on a 4-point scale (0 = *Never*, 1 = *Rarely*, 2 = *Sometimes*, 3 = *Often*, 4 = *Almost always)*.	Competence (α = 0.85), Affiliation (α = 0.90), Reactivity (α = 0.90). Study sample: α = 0.84; α = 0.89; α = 0.92.

IECI. “Inventario de estrés cotidiano infantil” [Inventory of Daily Stress in Children] (Trianes et al., 2011)	*General stress* Health/psychosomatic problems Stress in school setting Stress in family environment	Self-report. 22 dichotomic items (e.g.,“I often feel bad: I get headaches, nausea...”), with Yes-No response option.	α = 0.81. Study sample (α = 0.73)

*α = Cronbach’s alpha.*

### Procedure

The study used a descriptive, correlational, and cross-sectional design. Firstly, a letter was sent to the selected schools, explaining the research project. With the headmasters who agreed to participate, we scheduled an interview in which we explained the project in more detail, and we handed out informed consent forms for parents and/or legal guardians. The members of the research team went to the schools and administered six assessment instruments to the participants, in two 50-min assessment sessions, on successive days. In addition, the teacher filled in another instrument with regard to each child. The study met the ethical values required in research with humans and received the favorable report of the Commission of Research Ethics of the University of the Basque Country (CEISH/266MR/2014).

### Data Analysis

Before calculating the correlations between childhood depression and adaptive and clinical variables, we determined possible sex differences, performing descriptive analysis (means and standard deviations) and analysis of variance with the score obtained in depressive symptoms (CDS-self-assessment). Taking into account the absence of sex differences, we calculated the Pearson correlation coefficients for the entire sample. We calculated the correlation between depressive symptoms and the rest of the variables under study. Subsequently, in order to identify variables that predict depression, we conducted stepwise multiple linear regression analysis, first entering the global dimensions and then the subdimensions.

## Results

### Childhood Depression: Relations to Adaptive and Clinical Variables

The results obtained in the analysis of variance according to sex showed that the mean scores in depressive symptoms were higher in boys (*M* = 139.80, *SD* = 32.55) than in girls (*M* = 136.5, *SD* = 31.75) but these differences were not statistically significant, *F*(1,418) = 1.08, *p* = 0.299. Therefore, we calculated the correlations for the entire sample.

Firstly, when analyzing the global dimensions (**Table 2**), we found positive correlations between self-assessed depression and clinical maladjustment, school maladjustment, emotional symptoms, emotional and behavioral problems (internalizing and externalizing), problem behaviors, emotional reactivity and childhood stress, as well as negative correlations with personal adaptation, global self-concept, social skills, and resilience.

**Table 2:** Pearson Coefficients correlation between self-assessed depression and adaptive and clinical variables.

	Depressive symptoms *r* (*p*)
BASC. Clinical Scales	
Negative attitude to school	0.30 (0.001)
Negative attitude to teachers	0.31 (0.001)
Atypicality	0.45 (0.001)
Locus of control	0.49 (0.001)
Social stress	0.53 (0.001)
Anxiety	0.53 (0.001)
Depression	0.48 (0.001)
Sense of incapacity	0.46 (0.001)
BASC. Adaptive scales
Interpersonal relations	–0.31 (0.001)
Relations with parents	–0.18 (0.001)
Self-esteem	–0.27 (0.001)
Self-Confidence	–0.25 (0.001)
BASC. Global Indices
Clinical Maladjustment	0.57 (0.001)
School Maladjustment	0.35 (0.001)
Personal Adaptation	–0.36 (0.001)
Emotional Symptoms Index	0.57 (0.001)
SPECI. Emotional/Behavior Problems (Teachers)
Withdrawal	0.12 (0.012)
Somatization	0.09 (0.044)
Anxiety	0.09 (0.050)
Infantile-dependence	0.08 (0.093)
Thought problems	0.10 (0.029)
Attention-hyperactivity	0.18 (0.007)
Disruptive behavior	0.02 (0.602)
Academic achievement	0.13 (0.007)
Depression	0.14 (0.004)
Violent behavior	0.02 (0.632)
SPECI. Global Scales	
Internalizing problems	0.16 (0.001)
Externalizing problems	0.15 (0.001)
Emotional and Behavioral Problems	0.19 (0.001)
CAG. Self-concept	
Physical self-concept	–0.36 (0.001)
Social self-concept	–0.40 (0.001)
Family self-concept	–0.45 (0.001)
Intellectual self-concept	–0.30 (0.001)
Personal self-concept	–0.49 (0.001)
Sense of control	–0.32 (0.001)
Global Self-concept	–0.53 (0.001)
SSIS. Social skills	
Positive Scales	
Communication	–0.24 (0.001)
Cooperation	–0.30 (0.001)
Assertiveness	–0.29 (0.001)
Responsibility	–0.38 (0.001)
Empathy	–0.16 (0.001)
Involvement/participation	–0.29 (0.001)
Self-control	–0.30 (0.001)
Negative scales	
Externalizing	0.31 (0.001)
Bullying	0.17 (0.001)
Inattention-hyperactivity	0.40 (0.001)
Internalizing	0.58 (0.001)
SSIS. Global Scales	
Social skills	–0.39 (0.001)
Problem Behaviors	0.50 (0.001)
RSCA. Resilience Scales	
Optimism	–0.43 (0.001)
Self-efficacy	–0.37 (0.001)
Adaptability	–0.28 (0.001)
Self-Confidence	–0.43 (0.001)
Support	–0.29 (0.001)
Comfort	–0.34 (0.001)
Tolerance	–0.32 (0.001)
Sensitivity	0.29 (0.001)
Recovery	0.22 (0.001)
Alteration	0.33 (0.001)
RSCA. Global Resilience Indices	
Sense of competence	–0.41 (0.001)
Sense of affiliation	–0.41 (0.001)
Emotional reactivity	0.33 (0.001)
IECI. Childhood stress	
Stress due to health/psychosomatic problems	0.43 (0.001)
Stress in school setting	0.43 (0.001)
Stress in family environment	0.42 (0.001)
IECI. General stress	0.54 (0.001)

Secondly, when analyzing the subdimensions, the Pearson correlation coefficients (**Table 2**) confirmed positive correlations between self-assessed depression and the following variables: (1) *Negative attitude to school* (feelings of dissatisfaction toward school); (2) *Negative attitude to teachers* (feelings of dislike toward teachers); (3) *Atypicality* (tendency to sudden mood changes, strange ideas, unusual experiences, obsessive thoughts and behaviors considered “weird”); (4) *External locus of control* (belief that the consequences of behavior are controlled by external events or other people); (5) *Social stress* (stress due to interactions with others); (6) *Anxiety* (feelings of nervousness, worry, and fear, tendency to feel overwhelmed by problems); (7) *Depression* (feelings of loneliness and sadness, inability to enjoy life); (8) *Sense of incapacity* (perceptions of not succeeding in school, difficulty to achieve goals and general incapacity); (9) *Internalizing problems assessed by teachers*, such as, *Withdrawal* (shyness, tendency to avoid contact with others, to be alone, not talking much, inhibited social behavior), *Somatization* (numerous physical complaints that do not allow students to work properly, such as headache, stomach ache, back and chest pains... without entirely justifiable medical causes), *Anxiety* (restlessness, nervousness, internal tension, insecurity, fear of problems), *Thought problems* (inappropriate, inconsistent reasoning), *Depression* (sadness, apathy, crying easily, lack of pleasure); (10) *Externalizing problems assessed by teachers* such as *Attention-hyperactivity* (difficulty concentrating and paying attention, easily distracted, active, impulsive, impatient in the face of difficulties, low frustration tolerance), and *Academic achievement* (below average age-appropriate academic achievement not due to intelligence, apathy, lack of motivation to study or to learn); (11) *Self-assessed problematic externalizing behaviors* (physical and verbal aggressive behavior), *Bullying* (aggressive behaviors toward peers), *Inattention-hyperactivity* (easily distracted, impulsivity, excess activity), and *internalizing behaviors* (anxiety, sadness, loneliness); (12) *Emotional reactivity* (sensitivity or intense reactions; recovery or capacity to return from a state of agitation to emotional balance; and *alteration*, occurring when balance is not achieved); and (13) *Childhood stress* in all the assessed dimensions (health/psychosomatic problems, school stress, family stress).

On another hand, the Pearson correlation coefficients (**Table 2**) confirmed negative correlations between depression and the following variables: (1) *Interpersonal relations* (perception of good social relations with peers); (2) *Relations with parents* (positive attitude toward parents and feeling loved); (3) *Self-esteem* (feelings of self-acceptance); (4) *Self-confidence* (confidence in own problem-solving capacity, independence, capacity to make own decisions); (5) *Self-concept* (physical, social, intellectual, family, personal, sense of control); (6) *Social skills* (communication, cooperation, assertiveness, responsibility, empathy, involvement/participation, self-control); and (7) *Resilience*, both regarding a *sense of competence* (optimism, self-efficacy, adaptability) and a *sense of affiliation* (trusting others, perception of social support in adverse situations, or feeling comfortable with others, and tolerance or belief that one can express one’s differences within a relationship).

Of the set of variables analyzed, no relations were found between depression and *child-dependent behavioral problems* (behaviors that are more appropriate in smaller children, dependence on adults, emotional immaturity…), *disruptive behavior* (disruptive behavior in the classroom, lack of discipline, disobedience, disturbing others or the class), and *violent behavior* (theft, threats, hitting, making fun, vandalism, cruelty to animals…).

### Predictor Variables of Childhood Depression

In order to identify the predictor variables of child depression, we performed stepwise multiple linear regression analysis, introducing the global dimensions (clinical maladjustment, school maladjustment, personal adjustment, index of emotional symptoms, externalizing problems, internalizing problems, global self-concept, social skills, problematic social behaviors, sense of competence, affiliation, emotional reactivity, and general stress), the results of which are presented in **Table 3**.

**Table 3:** Multiple regression analysis for global dimensions predictive of childhood depression.

	*R*	*R^2^*	*Δ R^2^*	*B*	Error	Constant	β	*t*
BASC. Clinical Maladjustment	0.566	0.321	0.312	0.410	0.049	36.83	0.328	7.19^∗∗∗^
CAG. Global self-concept	0.682	0.466	0.463	–0.412	0.076	170.65	–0.263	–5.42^∗∗∗^
IECI. Childhood stress	0.707	0.499	0.495	2.418	0.463	171.30	0.243	5.27^∗∗∗^
SSIS. Social skills	0.715	0.512	0.506	–0.297	0.098	178.24	–0.142	–3.03^∗∗^

*^∗∗^*p* < 0.01; ^∗∗∗^*p* < 0.001.*

The results (**Table 3**) revealed four significant variables: clinical maladjustment (β = 0.328), global self-concept (β = -0.263), general stress (β = 0.243), and social skills (β = -0.142). The percentages of explained variance (adjusted determination coefficients) for each of these predictor variables were of medium magnitude. These four variables, which account for 50.6% of the variance, were predictive of depression: high clinical maladjustment, low global self-concept, high level of general stress, and few social skills.

Subsequently, we performed linear regression analysis, introducing all the sub-dimensions, the results of which are presented in **Table 4**. The results revealed nine significant variables: internalizing problems (β = 0.250), family self-concept (β = -0.183), anxiety (β = 0.152), responsibility (β = -0.163), personal self-assessment (β = -0.130), social stress (β = 0.103), bullying (β = -0.122), health/psychosomatic problems (β = 0.111), and external locus of control (β = 0.112). The percentages of explained variance (adjusted determination coefficients) for each of the predictor variables were of medium to high magnitude. Nine variables, which account for 56.4% of the variance, were predictive of depression: many internalizing problems, low family self-concept, high level of anxiety, low responsibility, low personal self-assessment, high social stress, few behaviors of bullying perpetration, many health/psychosomatic problems, and external locus of control.

**Table 4:** Multiple regression analysis for sub-dimensions predictive of childhood depression.

	*R*	*R^2^*	*Δ R^2^*	*B*	Error	Constant	β	*t*
SSIS. Internalizing problems	0.581	0.337	0.336	1.502	0.272	107.98	0.250	5.53^∗∗∗^
CAG. Family self-concept	0.653	0.426	0.423	–1.255	0.273	182.90	–0.183	–4.60^∗∗∗^
BASC. Anxiety	0.705	0.497	0.493	1.254	0.397	180.37	0.152	3.51^∗∗^
SSIS. Responsibility	0.724	0.523	0.519	–1.789	0.406	200.19	–0.163	–4.41^∗∗∗^
CAG. Personal self-assessment	0.733	0.537	0.531	–1.060	0.331	226.31	–0.130	–3.20^∗∗∗^
BASC. Social stress	0.740	0.548	0.541	1.606	0.777	219.36	0.103	2.06^∗^
SSIS. Bullying	0.747	0.558	0.550	–1.634	0.486	223.41	–0.122	–3.36^∗∗∗^
IECI. Health/Psychosomatic	0.753	0.567	0.558	2.401	0.838	221.73	0.111	2.86^∗∗^
BASC. Locus of control	0.757	0.573	0.564	1.190	0.500	218.09	0.112	2.38^∗^

*^∗^*p* < 0.05; ^∗∗^*p* < 0.01; ^∗∗∗^*p* < 0.001.*

## Discussion

The study aimed to analyze the relationship between childhood depression and a broad range of adaptive and clinical variables, as well as to identify variables that predict depression.

Firstly, the results showed that children with high scores on self-assessed symptoms of depression were more likely to have had *high clinical maladjustment* (anxiety, atypicality, external locus of control), *school maladjustment* (negative attitude toward school and teachers), *emotional symptoms* (anxiety, negative interpersonal relationships, low self-esteem, social stress, sadness-loneliness, sense of incapacity), *many emotional and internalizing and externalizing behavior problems* (withdrawal, somatization, anxiety, thought problems, attention-hyperactivity, low academic performance), *many problem behaviors* (aggressiveness, inattentive-hyperactivity, anxiety, sadness, loneliness), *high emotional reactivity* (vulnerability, agitation, hypersensitivity, alteration, emotional imbalance), and *high childhood stress* (many health/psychosomatic problems, physical symptoms without medical justification, high school stress related to problems with teachers, peers, and poor academic achievement, and high family stress associated with the perception of a lack of parental affection, perceived loneliness at home, perception of squabbles among siblings and high parental demand). The results pointing in the same direction as other studies that found low academic achievement (Wang et al., 2016; Jaureguizar et al., 2017) and health and adaptation problems (Wang et al., 2016) in depressed children.

Secondly, the results suggest that children with high scores on self-assessed symptoms of depression are more likely to have *low personal adjustment* (shown in difficulties in interpersonal relationships, in relationships with parents, low self-confidence, and low self-esteem), *low global self-concept* (physical, social, intellectual, family, personal...), *poor social skills* (low level of communication, cooperation, assertiveness, responsibility, empathy, involvement/participation, self-control), *low resilience*, that is, *low sense of competence* (low level of optimism, self-efficacy, adaptability) and of *affiliation* (low trust, low social support, feelings of discomfort, and low tolerance for difficulties). The data validate other studies that have shown connections between depressive symptoms and problems in peer relationships (Cole, 1991; Kochenderfer and Ladd, 1996; Brendgen et al., 2002; Katz et al., 2011; Bernaras et al., 2013; Hames et al., 2013), low self-esteem (Orth et al., 2008; Bos et al., 2010), and low resilience (Geng-Feng et al., 2016).

Thirdly, the regression analysis yielded four predictor variables of child depression that account for 50.6% of the variance: *high clinical maladjustment, low global self-concept, high level of general stress*, and *poor social skills*. In addition, when introducing all the sub-dimensions, we confirmed as predictors nine variables that explain 56.4% of the variance: *many internalizing problems* (anxiety, sadness, loneliness); *low family self-concept* (the family provides a low level of satisfaction); *high level of anxiety* (nervousness, worry, fear, tendency to feel overwhelmed by problems); *low responsibility* (low interest in school work), *low personal self-assessment*, (low global rating as a person), *high social stress* (due to interactions with others), *few behaviors of peer bullying* (as perpetrator), *health/psychosomatic problems* (physical problems, headaches, stomach ache…), and *external locus of control* (attribution of consequences to external factors). Hence, the results confirm the predictions and ratify studies that have found that anxiety (Kovacs and López-Durán, 2010), health and adaptation problems, problems in interpersonal relationships (Wang et al., 2016), poor social skills (Reinfjell et al., 2016), and low self-esteem (Babore et al., 2016) predict childhood depression.

Among the limitations of the study, we note the intentional selection of the sample. Therefore, future studies should use representative samples taken from educational contexts, and also samples of children who consult a psychologist for childhood depression at these early ages. Moreover, we recommend that future research should also obtain information from the parents because, in this study, we only obtained information about self-reported depressive symptoms and symptoms reported by teachers. Finally, as a limitation of this work, we note that the data are correlational, so they contribute little to the causal link between these variables. The methodology used is not experimental, so it does not allow us to categorically rule out the effect of third variables (for example, environmental factors…). More investigation with experimental or quasi-experimental designs would help to better determine the existence of connections among these variables.

Despite the limitations, the study makes a significant contribution, providing evidence of the significant connection of childhood depression with thought problems (inappropriate, incoherent reasoning, strange thoughts...), problems of attention-hyperactivity (difficulty concentrating and paying attention, easily distracted, poor task performance, impulsivity, low frustration tolerance...), emotional reactivity (vulnerability, low resilience, high agitation, alteration, emotional imbalance, lack of control...), and high level of general stress (social, school, family...). Few studies have identified predictive variables of child depression, and this work has identified as predictors a high level of general stress (social, school, family...), many health/psychosomatic problems (worries and physical problems that lead to frequent visits to the doctor), and external locus of control (external attribution of the consequences of behaviors).

These findings have relevant practical implications for the design of programs of prevention and psychological treatments for childhood depression. They mainly emphasize the importance of including in interventions to prevent/reduce childhood depression some activities aimed at: (1) *reducing childhood stress* in all contexts (social, school and family), for example, incorporating relaxation activities, cognitive training techniques to control negative thoughts of that generate stress..., which would have positive results in reducing anxiety and problems of attention-hyperactivity; (2) *enhancing self-esteem*, and feelings of self-acceptance; (3) *developing social skills*, the ability to integrate socially, communicate, cooperate, be assertive, empathetic, participatory, self-controlled..., because the increase in social competences will facilitate social interaction, thereby avoiding the situation of isolation and exclusion that many depressed children suffer; (4) *promoting internal locus of control* of life situations and their consequences; and (5) *stimulating resilience*, that is, optimism, self- efficacy, adaptability, self-confidence, feeling comfortable..., a positive factor which helps to cope with adversity and develop good personal adaptation. Resilience is shown to be a relevant goal to be included in the treatment and prevention of childhood depression.

In the educational context, the results advocate the implementation of programs of socio-emotional development, for example, programs of cooperative play and emotional intelligence, which can promote the development of social and emotional competences that are inversely related to childhood depression. In the clinical setting, the results suggest different goals and therapeutic strategies, such as: (1) teaching techniques to control anxiety (relaxation), (2) using behavioral techniques to develop social/prosocial skills, giving opportunities to practice/try out these behaviors and strengthening them; (3) using cognitive techniques to influence cognitive processes (attributions, expectations, problem-solving strategies and skills, control of negative thoughts...), for example, cognitive restructuring to change irrational beliefs to other more adaptive ways of thinking, self-instruction training (identifying internal negative stimuli, learning how to use reinforcing self-affirmations, positive self-appraisals...); and (4) using emotional techniques such as drawing and playing to facilitate the expression and constructive management of emotions (Garaigordobil et al., 1996; Garaigordobil, 1999, 2003; Del Barrio and Carrasco, 2013).

As has been highlighted in several studies, the prevalence of depression is worthy of consideration, as it is a public health problem that requires multidirectional intervention (family, school, clinic...). Taking into account the findings of the present study, we underline the importance of identifying early symptoms of childhood depression and suggest the implementation of educational programs that promote socio-emotional competences, and the systematized use of evidence-based, efficacious clinical treatments for childhood depression. Intervening in childhood depression will have a positive effect on the emergence of depression during adolescence and adulthood, and their concomitant severe consequences.

## Author Contributions

All authors listed, have made substantial, direct and intellectual contribution to the work, and approved it for publication.

## Conflict of Interest Statement

The authors declare that the research was conducted in the absence of any commercial or financial relationships that could be construed as a potential conflict of interest.

## References

[B1] American Psychiatric Association [APA] (2014). *Manual Diagnóstico y Estadístico de los Trastornos Mentales [Diagnostic and Statistical Manual of Mental Disorders] DSM-5.* Madrid: Panamericana.

[B2] Babore A. Trumello C. Candelori C. Paciello M. Cerniglia L. (2016). Depressive symptoms, self-esteem and perceived parent–child relationship in early adolescence. *Front. Psychol.* 7:982 10.3389/fpsyg.2016.00982 PMC4923143 27445941

[B3] Bernaras E. Jaureguizar J. Soroa M. Ibabe I. de las Cuevas M. C. (2013). Evaluación de la sintomatología depresiva en el contexto escolar y variables asociadas [Evaluation of Depressive Symptomatology and the Related Variables in the School Context]. *An. Psicol.* 29 131–140. 10.6018/analesps.29.1.137831

[B4] Bos A. E. R. Huijding J. Muris P. Vogel L. R. R. Biesheuvel J. (2010). Global, contingent and implicit self-esteem and psychopathological symptoms in adolescents. *Pers. Individ. Dif.* 48 311–316. 10.1016/j.paid.2009.10.025

[B5] Brendgen M. Vitaro F. Turgeon L. Poulin F. (2002). Assessing aggressive and depressed children’s social relations with classmates and friends: a matter of perspective. *J. Abnorm. Child Psychol.* 30 609–624. 10.1023/A:1020863730902 12481975

[B6] Cole D. A. (1991). Preliminary support for a competency-based model of depression in children. *J. Abnorm. Child Psychol.* 100 181–190. 10.1037/0021-843X.100.2.181 2040769

[B7] Colman I. Zeng Y. McMartin S. E. Naicker K. Ataullahjan A. Weeks M. (2014). Protective factors against depression during the transition from adolescence to adulthood: findings from a national Canadian cohort. *Prev. Med.* 65 28–32. 10.1016/j.ypmed.2014.04.008 24732721

[B8] Del Barrio V. (2000). *La Depresión Infantil. Factores de Riesgo y Posibles Soluciones [Child Depression. Risk Factors and Possible Solutions].* Málaga: Aljibe.

[B9] Del Barrio V. Carrasco M. A. (2013). *Depresión en Niños y Adolescentes [Depression in Children and Adolescents].* Madrid: Síntesis.

[B10] Garaigordobil M. (1999). Assessment of a cooperative-creative program of assertive behavior and self-concept. *Span. J. Psychol.* 2 3–10. 10.1017/S1138741600005400 11757258

[B11] Garaigordobil M. (2003). *Programa Juego 8-10 Años. Juegos Cooperativos y Creativos Para Grupos de Niños de 8 a 10 Años [Play Program for 8 to 10-Year-Olds.* Cooperative and Creative Games for Groups of Children Aged 8 to 10] Vol. 3 Madrid: Pirámide

[B12] Garaigordobil M. Maganto C. (2012). *SPECI: Screening de Problemas Emocionales y de Conducta Infantil [Screening of Emotional and Behavioral Problems during Childhood].* Madrid: TEA.

[B13] Garaigordobil M. Maganto C. Etxeberria J. (1996). Effects of a cooperative game program on socio-affective relationships and group cooperation capacity. *Eur. J. Psychol. Assess.* 12 140–151. 10.1027/1015-5759.12.2.141

[B14] García B. (2001). *CAG. Cuestionario de Autoconcepto: Manual [Self-Concept Questionnaire: Handbook].* Madrid: EOS.

[B15] Geng-Feng N. Xiao-Jun S. Yuan T. Cui-Ying F. Zong-Kui Z. (2016). Resilience moderates the relationship between ostracism and depression among Chinese adolescents. *Pers. Individ. Dif.* 99 77–80. 10.1016/j.paid.2016.04.059

[B16] González J. Fernández S. Pérez E. Santamaría P. (2004). *BASC. Sistema de Evaluación de la Conducta en Niños y Adolescentes (Adaptación Española) [Spanish Adaptation of the Behavior Assessment System for Children].* Madrid: TEA ediciones.

[B17] Gresham F. M. Elliott S. N. (2008). *Social Skills Improvement System Rating Scales Manual.* Minneapolis, MN: NCS Pearson.

[B18] Hames J. L. Hagan C. R. Joiner T. E. (2013). Interpersonal processes in depression. *Annu. Rev. Clin. Psychol.* 9 355–377. 10.1146/annurev-clinpsy-050212-185553 23297787

[B19] Jaureguizar J. Bernaras E. Garaigordobil M. (2017). Child depression: prevalence and comparison between self-reports and teacher reports. *Span. J. Psychol.* 20:E17 10.1017/sjp.2017.14 28224880

[B20] Katz S. J. Conway C. C. Hammen C. L. Brennan P. A. Najman J. M. (2011). Childhood social withdrawal, interpersonal impairment, and young adult depression: a mediational model. *J. Abnorm. Child Psychol.* 39 1227–1238. 10.1007/s10802-011-9537-z 21744059 PMC3384496

[B21] Kochenderfer B. J. Ladd G. W. (1996). Peer victimization: cause or consequence of school maladjustment? *Child Dev.* 67 1305–1317. 10.1111/j.1467-8624.1996.tb01797.x 8890485

[B22] Kosterman R. Hawkins J. D. Mason W. A. Herrenkohl T. I. Lengua L. J. Mccauley E. (2010). Assessment of behavior problems in childhood and adolescence as predictors of early adult depression. *J. Psychopathol. Behav. Assess.* 32 118–127. 10.1007/s10862-009-9138-0 PMC2850104 20383270

[B23] Kovacs M. López-Durán N. (2010). Prodromal symptoms and atypical affectivity as predictors of major depression in juveniles: implications for prevention. *J. Child Psychol. Psychiatry* 51 472–496. 10.1111/j.1469-7610.2010.02230.x 20202041 PMC2921595

[B24] Lamers F. van Oppen P. Comijs H. Smit J. Spinhoven P. van Balkom A. (2011). Comorbidity patterns of anxiety and depressive disorders in a large cohort study: the Netherlands study of depression and anxiety (NESDA). *J. Clin. Psychiatry* 72 341–348. 10.4088/JCP.10m06176blu 21294994

[B25] Lang M. Tisher M. (1978). *Children’s Depression Scale.* Camberwell, VIC: Australian Council for Educational Research.

[B26] Orth U. Robins R. W. Roberts B. W. (2008). Low self-esteem prospectively predicts depression in adolescence and young adulthood. *J. Pers. Soc. Psychol.* 95 695–708. 10.1037/0022-3514.95.3.695 18729703

[B27] Poole J. C. Dobson K. S. Pusch D. (2017). Childhood adversity and adult depression: the protective role of psychological resilience. *Child Abuse Negl.* 64 89–100. 10.1016/j.chiabu.2016.12.012 28056359

[B28] Prince-Embury S. (2008). *Resiliency Scales for Children & Adolescents (RSCA).* San Antonio, TX: Pearson.

[B29] Qualter P. Brown S. L. Munn P. Rotenberg K. J. (2010). Childhood loneliness as a predictor of adolescent depressive symptoms: an 8-year longitudinal study. *Eur. Child Adolesc. Psychiatry* 19 493–501. 10.1007/s00787-009-0059-y 19777287

[B30] Reinfjell T. Kårstad S. B. Berg-Nielsen T. Luby J. L. Wichstrøm L. (2016). Predictors of change in depressive symptoms from preschool to first grade. *Dev. Psychopathol.* 28 1517–1530. 10.1017/S0954579415001170 26645908

[B31] Reynolds C. R. Kamphaus R. W. (1992). *Behavior Assessment System for Children (BASC).* Circle Pines, MN: American Guidance Services.

[B32] Seisdedos N. (2003). *Cuestionario de Depresión para Niños. Manual. (7^a^ Edn. Adaptación española del CDS) [Depression Questionnaire for Children. Manual. Spanish adaptation of the CDS]* 7th Edn Madrid: TEA.

[B33] Trianes M. V. Blanca M. J. Fernández-Baena F. J. Escobar M. Maldonado E. F. (2011). *IECI. Inventario de Estrés Cotidiano Infantil [Inventory of Daily Stress in Children].* Madrid: TEA.

[B34] Wang L. Feng Z. Yang G. Yang Y. Wang K. Dai O. (2016). Depressive symptoms among children and adolescents in western China: an epidemiological survey of prevalence and correlates. *Psychiatry Res.* 246 267–274. 10.1016/j.psychres.2016.09.050 27741479

[B35] World Health Organization [WHO] (2016). *Depression.* Available at: http://www.who.int/mediacentre/factsheets/fs369/es/ [accessed February 2017].

[B36] Wu Y. L. Zhao X. Li Y. F. Ding X. X. Yang H. Y. Bi P. (2016). The risk and protective factors in the development of childhood social anxiety symptoms among Chinese children. *Psychiatry Res.* 240 103–109. 10.1016/j.psychres.2015.08.046 27092863

